# The Warwick patellofemoral arthroplasty trial: a randomised clinical trial of total knee arthroplasty versus patellofemoral arthroplasty in patients with severe arthritis of the patellofemoral joint

**DOI:** 10.1186/1471-2474-12-265

**Published:** 2011-11-23

**Authors:** Michelle Odumenya, Katie McGuinness, Juul Achten, Nick Parsons, Tim Spalding, Matthew Costa

**Affiliations:** 1 Division of Health Sciences, Warwick Medical School, University of Warwick, Clifford Bridge Road, Coventry, CV2 2DX, United Kingdom; 2 University Hospitals Coventry & Warwickshire NHS Trust, Trauma & Orthopaedic Department, Clifford Bridge Road, Coventry, CV2 2DX, United Kingdom

## Abstract

**Background:**

Severe arthritis of the knee is a disabling condition, with over 50,000 knee replacements performed each year in the UK. Isolated patellofemoral joint arthritis occurs in over 10% of these patients with the treatment options being patellofemoral arthroplasty or total knee arthroplasty. Whilst many surgeons believe total knee arthroplasty is the 'gold standard' treatment for severe knee arthritis, patellofemoral arthroplasty has certain potential advantages. Primarily, because this operation allows the patient to keep the majority of their own knee joint; preserving bone-stock and the patients' own ligaments. Patellofemoral arthroplasty has also been recognised as a less 'invasive' operation than primary total knee arthroplasty, facilitating a more rapid recovery. There are currently no published results of randomised clinical trials comparing the two arthroplasty techniques. The primary objective of the current study is to assess whether there is a difference in functional knee scores and quality of life outcome assessments at one year post-operation between patellofemoral arthroplasty and total knee arthroplasty. The secondary objective is to assess the complication rates for both procedures.

**Methods/design:**

Patients who are deemed suitable, by an Orthopaedic Consultant, for patellofemoral arthroplasty and medically fit for surgery are eligible to take part in this trial. The consenting patients will be randomised in a 1:1 allocation to a total knee or patellofemoral arthroplasty. The randomisation sequence will be computer generated and administered by a central independent randomisation service. Following consent, all participants will have their knee function, quality of life and physical activity level assessed through questionnaires. The assigned surgery will then be performed using the preferred technique and implant of the operating surgeon. The first post-operative assessments will take place at six weeks, followed by further assessments at 3, 6 and 12 months. At each assessment time point all complications will be recorded. In addition, community and social care services usage will be collected using a patient questionnaire at 3, 6 & 12 months. The patients will then be sent an annual postal questionnaire. The questionnaire will ask about any problems, knee pain and function following their knee arthroplasty to monitor long-term function and failure rates.

**Discussion:**

This trial is expected to deliver results in early 2013.

**Trial Registration:**

ISRCTN: ISRCTN34863373

UKCRN portfolio ID 6847

## Background

### Isolated patellofemoral arthritis and treatment options

Severe arthritis of the knee is a common and disabling condition. Over 50,000 patients require a knee arthroplasty each year in the UK at an estimated cost of 300 million pounds. Arthritis confined to the patellofemoral joint (the articulation between the patella and the trochlear groove of the femur) occurs in over 10% of these patients. The average age of those affected by isolated arthritis of the patellofemoral joint is significantly lower than those with severe generalised arthritis [1]. Commonly, patients complain of anterior knee pain which limits their daily activities and in severe cases patients are unable to continue in employment. Initially, these symptoms can be successfully treated non-operatively. However, in severe debilitating cases the efficacy of these non-surgical modalities is minimal and the only alternative treatment is surgery. The operative treatment options for this disease include arthroscopic procedures, patellectomy (removal of the knee-cap), Total Knee Arthroplasty (TKA) and more recently, Patellofemoral Arthroplasty (PFA). Arthroscopic surgery is seldom beneficial in severe disease and patellectomy often leads to poor long-term function, therefore the choice for patients is usually between TKA and PFA.

Whilst many surgeons believe TKA is the 'gold standard' treatment for severe knee arthritis, PFA has certain potential advantages. Primarily, this operation allows the patient to keep the majority of their own knee joint; preserving bone-stock and the patients' own ligaments. PFA has also been recognised as a less 'invasive' operation than TKA, facilitating a more rapid recovery [2]. Despite these perceived advantages, early PFA designs yielded less than satisfactory results due to residual patella mal-alignment, wear of polyethylene and failure secondary to disease progression in the other parts of the joint [3]. More recent studies, however, have shown significantly better results with PFA. This is due to improved implants, an appreciation of the need to balance the soft tissues and more appropriate patient selection when considering PFA [4].

In a retrospective case series study, Cartier et al. [5] reported excellent functional outcome in 77% of patients at a mean of 10 years follow-up. These findings substantiate those of an earlier retrospective consecutive case study, where good or excellent functional results were reported, using the Knee Society Score (KSS), in 86% of 'residual' cases at 17 years [6]. Most recently, Odumenya et al. [7] and Stark et al [8] have reported excellent functional outcomes with 100% survivorship at 5 and 2 years follow-up, respectively. Therefore, there is some evidence to suggest that PFA provides positive results for patients with isolated patellofemoral arthritis. It is however not clear how the latest generation of PFA compare with the excellent long-term results of Total Knee Arthroplasty [9]. The majority of the remaining literature consists of case series with short-term results and includes only a small number of studies on the use of TKA for primarily severe patellofemoral arthritis. Mont et al. [10] reported 97% good or excellent clinical results using the KSS at a mean follow-up of over 6 years. These results are in agreement with the findings of Laskin et al. [11] who reported on forty-two patients managed with TKA for primary patellofemoral arthritis. This study compared the use of TKA in patients with patellofemoral arthritis and tricompartmental arthritis. They concluded that the results of TKA in the patellofemoral subgroup were superior to those patients with tricompartmental arthritis. However, these inferences are uninformative when deciding between TKA and PFA for the treatment of isolated patellofemoral arthritis.

There are currently no published results of randomised clinical trials comparing the two arthroplasty techniques. At present the only registered study (ISRCTN22478626) directly comparing TKA and PFA was not performed. We therefore propose the first trial comparing TKA with PFA to provide surgeons and patients with accurate information regarding knee function. The null hypothesis for this trial is that there is no difference in functional score (WOMAC - Western Ontario and McMaster Universities Osteoarthritis Index score) at one year post-operation between Total Knee Arthroplasty and Patellofemoral Arthroplasty.

## Methods/Design

### Design

This is a two-arm, double-blind, randomised clinical trial performed in a single centre (University Hospitals Coventry and Warwickshire NHS Trust) in the United Kingdom. The participant and assessor (research associate) are both blinded to the treatment allocation throughout the entire study. This study has been reviewed by the Coventry Research Ethics Committee under reference number 09/H1210/9. The study was approved on the 3^rd ^of March 2009. The research carried out is in compliance with the Helsinki Declaration.

### Study participants

#### Inclusion criteria

The eligibility criteria for participation in this study is that the patient is medically fit for an operation and has severe isolated patellofemoral osteoarthritis of the knee deemed suitable for a patellofemoral arthroplasty by the responsible consultant orthopaedic surgeon. It is accepted that all patients deemed suitable for PFA are also suitable for TKA. These broad eligibility criteria should ensure that the results of the study can readily be generalised to the wider population of patients with severe isolated patellofemoral osteoarthritis.

#### Exclusion criteria

Contra-indications to surgery include patients with tibiofemoral osteoarthritis of the knee and those unfit for surgery, defined as: (i) severe cardiac impairment, e.g. heart or valve replacement, arrhythmia, previous myocardial infarction, (ii) severe respiratory impairment, e.g. chronic obstructive pulmonary disease, asthma that has required hospital admission, or (iii) any other systemic medical condition that would produce a specific contraindication to a general anaesthetic. There is no fixed age range.

In addition, patients who are unable to adhere to trial procedures or complete questionnaires, for reasons such as dementia or intravenous drug use, will be excluded.

If a recruited patient requires contra-lateral knee arthroplasty surgery during the trial period, this second knee will not be included in the study (i.e. it will not be randomised) since the result of this intervention would not be independent of the first intervention.

#### Recruitment

Patients will be recruited from orthopaedic clinics at University Hospitals Coventry and Warwickshire NHS Trust. Upon identification of an eligible patient by the orthopaedic consultant, the patient will be referred to the research associate present in the clinic. The research associate will provide the patient with the patient information sheet and verbally explain what the trial entails. The patient will then be given the opportunity to discuss any issues with the research associate, their consultant as well as members of their family and friends.

#### Consent

Informed consent will be obtained by the research associate, after allowing sufficient time for the patient to consider their decision and ask questions about the trial. Any new information that arises during the trial that may affect participants' willingness to take part will be reviewed by the Trial Steering Committee, and, if necessary, communicated to all participants by the trial coordinator.

If the participant decides to withdraw, their care will continue as normal for the hospital. If the participant decides to continue in the study they will be asked sign an updated consent form.

#### Post randomisation withdrawals

Patients may withdraw from the trial at any time without discrimination. If patients decide to have the treatment to which they were not randomised, they will be followed-up where feasible and data collected as per the protocol until completion of the trial. The primary analysis will be on an intention-to-treat basis with a secondary per-protocol analysis. If patients have neither treatment during the course of the trial, they will be followed up in the same way as the other patients in the trial and included in the intention-to-treat analysis.

### The Study Intervention

The two treatments in this study are patellofemoral arthroplasty and total knee arthroplasty. In this pragmatic trial, each patient will undergo the allocated surgery according to the preferred technique and preferred implants of the operating surgeon.

In summary, the details of the surgery will be left entirely to the discretion of the surgeon to ensure that the results of the trial can be generalised to as wide a group of patients and surgeons as possible.

#### Patellofemoral Arthroplasty

**Figure 1:**
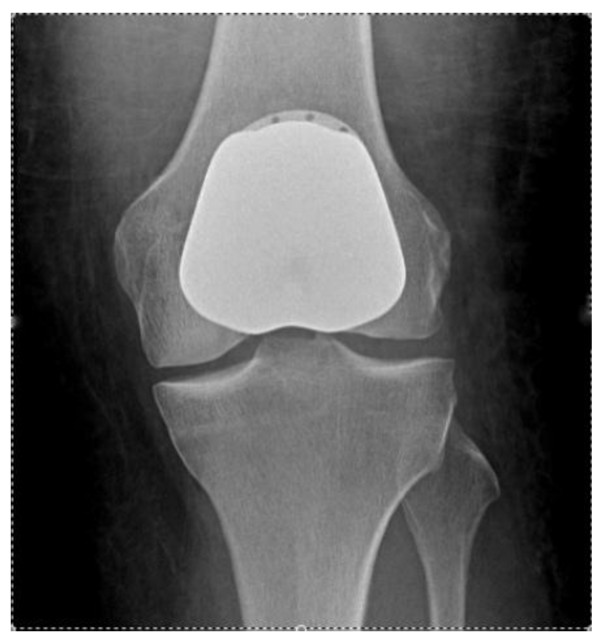
**AP radiograph showing a Patellofemoral Arthroplasty**.

Patellofemoral joint arthroplasty involves replacement of the articular surfaces of the femoral trochlear groove with a contoured metal implant and, usually, the under-surface of the patella with a plastic 'button'. Both articulating areas are resected using jigs and clamps respectively. The implants are then cemented in place using bone cement (see Figure 1). The native tibiofemoral joint remains in situ.

#### Total Knee Arthroplasty

**Figure 2:**
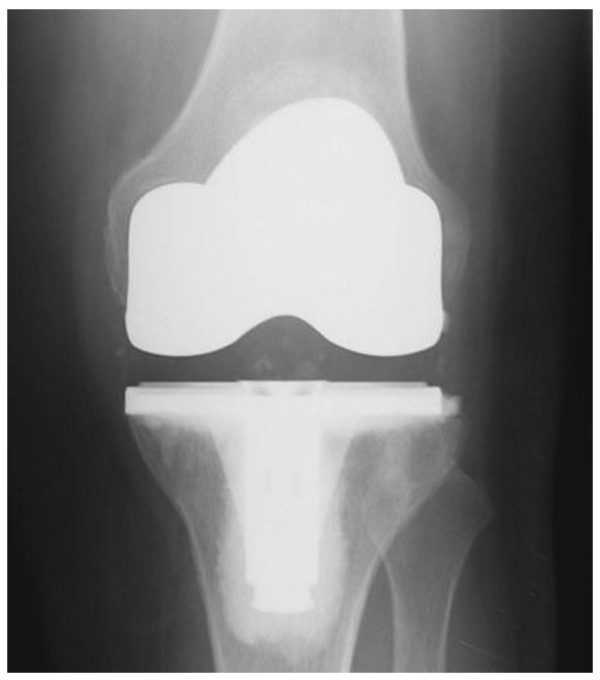
**AP radiograph showing a Total Knee Arthroplasty**.

In total knee arthroplasty, the tibiofemoral articulation is also replaced. The top of the tibia is resurfaced with a flat metal plate with a plastic 'spacer' on top. The upper part of the knee joint, the femoral condylar articulating surface, is replaced with a contoured metal implant that fits around it and includes the trochlear groove. The patella is usually resurfaced with a plastic button similar to that used in the PFA. All the implants are cemented to bone (see Figure 2).

#### Rehabilitation

The same standard rehabilitation program will be implemented for all patients, as outlined in the University Hospitals Coventry and Warwickshire NHS Trust 'Knee Replacement: A Guide for Patients' booklet. This includes early exercises, precautions to be followed for the first three months, how to perform functional activities and advanced exercises.

#### Follow-up

Patients will continue in routine clinical follow-up, according to their surgeon's practice. For this trial, the primary outcome point will be at one year, with other scheduled assessments at 6 weeks, 3 months and 6 months post operation. At all these assessment time points patients will come to the clinic to be reviewed by the research associate, and if necessary an orthopaedic surgeon. Thereafter, an annual postal questionnaire will be sent to each patient including the Oxford Knee Score, Euroqol and an additional treatment questionnaire to monitor long term function and failure rates.

In this study, we will use techniques common in long term cohort studies to ensure minimum loss to follow-up, such as collection of multiple contact addresses and telephone numbers, mobile telephone numbers and email addresses. Considerable efforts will be made by the trial team to keep in touch with patients. Using these techniques, with only 64 participants to track, we are confident that loss will not exceed 10%. In the event of a participant being lost to one year follow-up, we will consider, on the advice of the trial statistician, imputing missing primary outcome data from interim scores.

A system of reminders will be instituted to ensure that return to clinic at three, six and twelve months is as complete as possible. The research associate will phone participants to make an appointment and after two weeks non-response a letter will be sent out to the patient. The letter will be followed up by phone call after 1-2 weeks. If there is no response from participant, they will be classified as a 'non-responder'.

### Study objectives

There are two main objectives of this randomised clinical trial:

1. To quantify and draw inferences on observed differences in primary and secondary outcomes measures between the trial treatment groups at one year post-operation.

2. To determine the complication rate of Patellofemoral Arthroplasty versus Total Knee Arthroplasty at one year post-operation.

### Outcome measures

The primary outcome measure is: ***WOMAC (Western Ontario and McMaster Universities) Osteoarthritis Index score***. This is a validated questionnaire, which is self-administered (filled out by the patient). It consists of 24 items (5 for pain, 2 for stiffness and 17 for physical function), all related to daily tasks, which are directly influenced by poor function resulting from osteoarthritis. The minimum (best) score attainable is 0 and the maximum (worst) score is 96 [12].

Five secondary outcome measures will be used in this trial: (i) ***Oxford Knee Score (OKS)***: This is a self-administered, validated knee arthroplasty functional outcome measure consisting of 12 items related to daily activities. The score ranges from 12 to 60, where 12 represents the best outcome and 60 represents the worst outcome [13]., (ii) ***American Knee Society Score (AKSS)***: This is also a validated knee function questionnaire, which is divided into two sections: knee score and function score. The knee score is comprised of assessments of range of motion, stability, alignment, and muscle power of the knee. The function score includes an assessment of daily tasks to determine functional ability. Both scores range from 0 to 100 points (100 points being the best score). The research associate fills out this questionnaire [14], (iii) ***Euroqol (EQ-5D)***: This validated quality of life (QoL) questionnaire consists of 5 domains related to daily activities, each with a 3-level answer possibility. The combination of answers leads to the QoL score. The patient completes this form [15], (iv) ***Disability Rating Index***: a self-administered, 12-item Visual Analogue Score (VAS) questionnaire assessing the patient's own rating of disability [16], (v) ***UCLA Physical Activity Questionnaire***: The University of California Los Angeles activity-level rating is a self-rating scale from level 0-complete inactivity to level 10- participation in impact sports [17]. All complications will be recorded throughout the duration of the trial.

### Sample size

Previous work has suggested that the WOMAC score is the most sensitive condition-specific tool for assessing interventions in knee osteoarthritis [12,18]. Angst et al. [19] successfully demonstrated the use of the 17-item functional section of the WOMAC score to determine sample size.

The sample size calculation was based on a two-arm parallel group design and an assumed approximate normal distribution for the primary outcome measure (WOMAC). The required number of patients in each arm of the trial is 29, based on an independent samples *t*-test and an assumed population standard deviation of 10.8 points [20] and a minimum clinically important difference of 8 points [19]; this gives a relatively large standard effect size of 0.74. The proposed number of patients will provide 80% power to detect a difference at the 5% level (PS, Power and Sample Size Calculation Software version 2.1 30th February 2003, available at http://biostat.mc.vanderbilt.edu/wiki/Main/PowerSampleSize). To allow for a 10% loss to follow-up we will aim to recruit 32 patients in each arm, giving a total of 64 patients.

### Randomisation

Those patients who consent to take part in the trial will be randomised in a 1:1 allocation to TKA or PFA. The randomisation sequence will be computer generated. An independent researcher will phone the secure centralised, telephone randomisation service. The treatment allocation will then be emailed to the medical secretary of the operating consultant by the randomisation service. The medical secretary will then enter the allocation on to the patient's operation booking form. This will ensure that the research associates collecting outcome data remain blind to the treatment allocation,

### Blinding

The patients will also be blind to their treatment allocation to allow for an unbiased comparison to be made between the two interventions. The surgeons will, of course, not be blind to the treatment but will take no part in the post-operative assessment of the patients.

### Statistical analysis

Standard statistical summaries (e.g. medians and ranges or means and variances, dependent on the assumed distribution of the outcomes) and graphical plots showing correlations will be presented for the primary outcome measure (WOMAC) and the secondary outcome measures. Baseline data will be summarized to check comparability between treatment arms, and to highlight any characteristic differences between those individuals in the study, those ineligible, and those eligible but withholding consent. The main analysis will investigate differences in the primary outcome measure, the WOMAC score, between the two treatment groups on an intention-to-treat basis at 12 months post-operation. The significance in responses between treatment groups will be formally assessed using an independent samples *t*-test; based on an assumed approximate normal distribution for this outcome measure. Tests will be two-sided and considered to provide evidence for a significant difference if p-values are less than 0.05 (5% significance level). Estimates of treatment effects will be presented with 95% confidence intervals. A linear regression analysis will also be undertaken that will incorporate terms that model the effects of patient age and gender in addition to the effects of the treatment groups (TKA and PFA). Results from the unadjusted and adjusted analyses will be presented graphically, together with diagnostic plots that check the underlying model assumptions. Depending on the nature of the data, various derived variables (e.g. logarithmic transformations of scores) may be created and analysed, in addition to the primary outcome. Although missing data is not expected to be a problem for this study, the nature and pattern of the missingness will be carefully considered -- including in particular whether data can be treated as missing completely at random (MCAR). If judged appropriate, missing data will be imputed, probably using the multiple imputation facilities available in R http://www.r-project.org/. A detailed statistical analysis plan (SAP) will be agreed with the Data Management Committee (DMC) at the start of the study. Any subsequent amendments to this initial SAP will be clearly stated and justified. Interim analyses will be performed only where directed by the DMC. The routine statistical analysis will be carried out using R http://www.r-project.org/.

This trial is expected to deliver results in early 2013.

## Abbreviations

PFA: Patellofemoral Arthroplasty; TKA: Total Knee Arthroplasty; WOMAC: Western Ontario and McMaster Universities Osteoarthritis Index Score.

## Competing interests

The authors declare that they have no competing interests. The department of trauma and orthopaedics at UHCW does not receive funding from manufacturers of total knee arthroplasty. UHCW has held research grants from manufacturers of both total hip arthroplasty and patellofemoral arthroplasty implants, but not in relation to this trial.

## Authors' contributions

MO developed the protocol, assisted in securing grant funding and is responsible for recruitment of trial patients. JA developed the protocol, assisted in securing grant funding and manages the running of the trial. NP developed the protocol, assisted in securing grant funding and is responsible for the statistical analysis of the trial. KM developed the protocol and manages the running of the trial. TS developed the protocol and is responsible for the recruitment of trial patients. MC developed the protocol, secured the grant funding, is responsible for the recruitment of trial patients, management of trial and has overall clinical responsibility for the conduct of the trial. All authors read and approved the final manuscript.

## Pre-publication history

The pre-publication history for this paper can be accessed here:

http://www.biomedcentral.com/1471-2474/12/265/prepub
